# Opposing Effects of PhoPQ and PmrAB on the Properties of *Salmonella enterica* serovar Typhimurium: Implications on Resistance to Antimicrobial Peptides

**DOI:** 10.1021/acs.biochem.1c00287

**Published:** 2021-09-22

**Authors:** Tal Shprung, Naiem Ahmad Wani, Miriam Wilmes, Maria Luisa Mangoni, Arkadi Bitler, Eyal Shimoni, Hans-Georg Sahl, Yechiel Shai

**Affiliations:** † Department of Biomolecular Sciences, The Weizmann Institute of Science, Rehovot 76100, Israel; ‡ Pharmaceutical Microbiology Section, Institute for Medical Microbiology, Immunology and Parasitology, University of Bonn, Sigmund-Freud-Strasse 25, D-53127 Bonn, Germany; § Department of Biochemical Sciences A. Rossi Fanelli, Faculty of Pharmacy and Medicine, Sapienza University of Rome, CU27, 00185 Roma, Italy; ∥ Department of Chemical Research Support, The Weizmann Institute of Science, Rehovot 76100, Israel

## Abstract

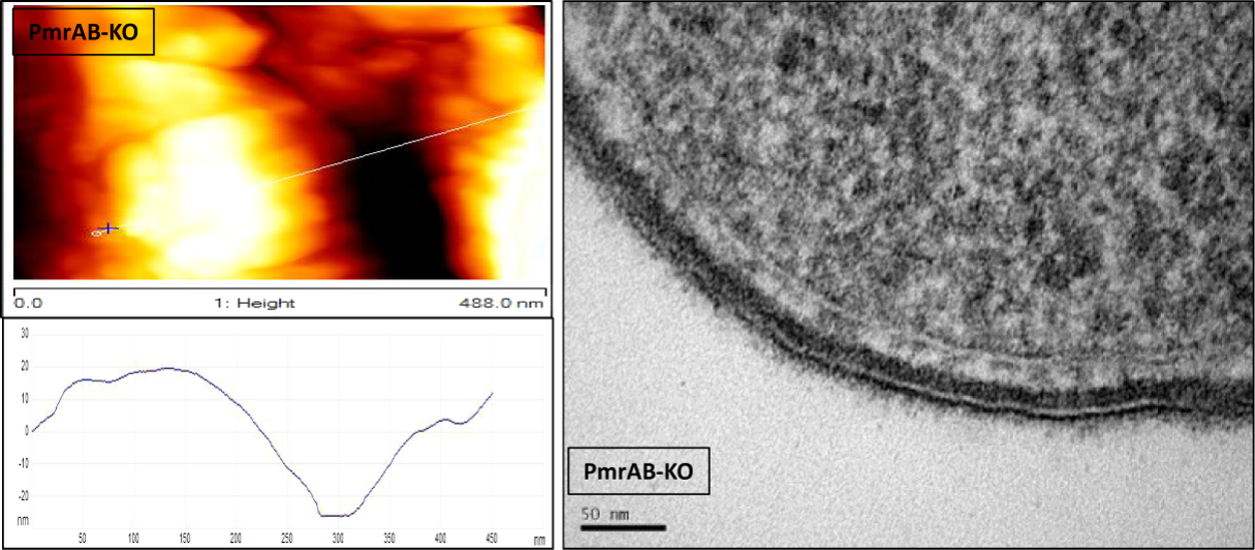

The increasing number of resistant bacteria is a major threat worldwide, leading to the search for new antibiotic agents. One of the leading strategies is the use of antimicrobial peptides (AMPs), cationic and hydrophobic innate immune defense peptides. A major target of AMPs is the bacterial membrane. Notably, accumulating data suggest that AMPs can activate the two-component systems (TCSs) of Gram-negative bacteria. These include PhoP-PhoQ (PhoPQ) and PmrA-PmrB (PmrAB), responsible for remodeling of the bacterial cell surface. To better understand this mechanism, we utilized bacteria deficient either in one system alone or in both and biophysical tools including fluorescence spectroscopy, single-cell atomic force microscopy, electron microscopy, and mass spectrometry (MoskowitzS. M.;Antimicrob. Agents Chemother.2012, 56, 1019−10302210622410.1128/AAC.05829-11PMC3264203; ChengH. Y.;J. Biomed. Sci.2010, 17, 602065397610.1186/1423-0127-17-60PMC2919465). Our data suggested that the two systems have opposing effects on the properties of *Salmonella enterica*. The knockout of PhoPQ made the bacteria more susceptible to AMPs by making the surface less rigid, more polarized, and permeable with a slightly more negatively charged cell wall. In addition, the periplasmic space is thinner. In contrast, the knockout of PmrAB did not affect its susceptibility, while it made the bacterial outer layer very rigid, less polarized, and less permeable than the other two mutants, with a negatively charged cell wall similar to the WT. Overall, the data suggest that the coexistence of systems with opposing effects on the biophysical properties of the bacteria contribute to their membrane flexibility, which, on the one hand, is important to accommodate changing environments and, on the other hand, may inhibit the development of meaningful resistance to AMPs.

## Introduction

The battle between humans and pathogenic microorganisms was thought^1,2^ to be developed once antibiotics were discovered. Unfortunately, the increasing number of resistant bacteria became a major threat worldwide, leading to the search for new antibiotic agents.^3−5^ We now encounter bacteria resistant to multiple antibiotics with different mechanisms of action.^6−8^ Therefore, there is an ongoing effort to develop novel antibiotics with new mechanisms of action. One of the leading strategies being investigated is the use of antimicrobial peptides (AMPs), which are cationic and hydrophobic peptides used by all types of living organisms as part of their innate immune system targeting the bacterial surface.^9−11^

AMPs rapidly neutralize a broad range of microbes, both Gram-negative and Gram-positive bacteria, mainly by binding and perturbing their cell envelope, which contains lipopolysaccharide (LPS) or lipoteichoic acid (LTA), respectively, as well as their cytoplasmic membranes.^12,13^ Therefore, studying the remodeling mechanisms of the outer surface is important to understand the interaction between bacteria and AMPs.^14^

Bacteria, being unicellular organisms, possess several sensory systems that are able to sense the environment and respond by activating different genes. Two such systems in Gram-negative bacteria are the two-component systems (TCSs) PhoP-PhoQ (PhoPQ) and PmrA-PmrB (PmrAB, BasRS in *Salmonella*).^15−17^ In these systems, a sensory protein/loop (PhoQ or PmrB, respectively), situated on the inner membrane, is activated by an environmental signal, which in turn phosphorylates a cytoplasmic protein (PhoP or PmrA, respectively) that is able to induce several genes. These systems respond to changes in the environmental concentrations of magnesium, iron, and aluminum ions, pH,^18−21^ and AMPs.^22−24^ It is suggested that AMPs bind to the acidic Mg^2+^ domain on the membrane receptor PhoQ.^25,26^ In addition, histidine-rich AMPs were suggested to act as chelators of magnesium ions, reducing Mg^2+^ concentration in the environment, causing PhoPQ activation.^23,27^

In *Salmonella enterica*, PmrAB, on the other hand, was not found to be directly activated by AMPs but by the PhoPQ-activated gene, pmrD, which is known to directly activate the PmrB response regulator, thereby activating PmrAB-regulated genes.^28,29^

Upon activation, PhoPQ and PmrAB induce many genes, some of which encode enzymes that modify the composition of the bacterial surface, including LPS,^30−33^ in addition to other functions that are involved in pathogenicity. For example, the enzymes encoded by pagP and pagL, which are induced by PhoPQ, modify the lipid-A of the LPS, while the products of pmrE and the pmrHJIFKLM operon, induced by PmrAB, modify its charge and composition.^34−36^ Yet, many of the genes induced by these systems have unknown functions.^36^ Because the purpose of these systems is to sense and prepare the bacteria for the changing environment, their combined action is believed to lead to the development of resistance to AMPs.^23,37^

Indeed, the relevance of these systems to the resistance of bacteria to AMPs was demonstrated when phoP-KO or pmrA-KO bacteria displayed modified susceptibility to some AMPs (particularly polymyxin B and polymyxin E) compared to the wild-type (WT).^38−40^ However, despite extensive studies, most of the work regarding these systems is of a genetic nature.^41−43^

Surprisingly, although PmrAB can be activated upon PhoPQ activation, we have found that each of these systems has an opposite effect on some aspects of bacterial biophysical properties and thus have a different effect on resistance depending on the AMP. This might suggest that each system confers resistance to a different kind of AMP, which may have implications on the correlation between the mechanism of action of AMP and how resistance is developed against it. Here, we investigated the contribution of the PhoPQ and PmrAB systems to resistance to AMPs in *Salmonella typhimurium* by utilizing a biophysical approach to study bacterial rigidity, morphology, modification of surface charge, membrane polarization, membrane permeation, and cell wall density. By constructing bacteria lacking PhoP alone, PmrAB alone, or both, we tried to shed light on the complex relationship between these two systems. Surprisingly, although PmrAB can be activated upon PhoPQ activation, we have found that each of these systems has an opposite effect on some aspects of bacterial biophysical properties, and each of the systems has a different effect on the resistance to different AMPs, which might suggest that these systems confer resistance to different kinds of AMPs, and it may imply to the mechanisms of actions and resistance of different AMPs.

## Materials and Methods

Rink amide 4-methylbenzhydrylamine (MBHA) resin and *N*-(9-fluorenyl)methoxycarbonyl (Fmoc)-amino acids were purchased from Calbiochem-Novabiochem. Other reagents used for peptide synthesis included trifluoroacetic acid (TFA, Sigma-Aldrich), *N*,*N*-diisopropylethylamine (DIEA, Sigma-Aldrich), dichloromethane (DCM, peptide synthesis grade, Bio-Lab), dimethylformamide (DMF, peptide synthesis grade, Bio-Lab), and hydroxybenzotriazole (HOBT) and 2-(1*H*-benzotriazol-1-yl)-1,1,3,3-tetramethyluronium hexafluorophosphate (HBTU) (peptide synthesis grade, Bio-Lab). Three *S. enterica* serovar Typhimurium strains ATCC 14028 (WT), the phoP-knockout derivative,^44^ a gift from Prof. Shoshi Altuvia’s lab (the Hebrew University, Jerusalem), and a PmrAB knockout were used in the present study. Kanamycin, tetracycline, and chloramphenicol were purchased from Sigma (catalog no. P1004, K-1377, T-3383, C0378, respectively). The media used were lysogeny broth (LB, containing 20 g/L LB broth, Conda), SOC (26.6 g/L SOB, Conda, supplemented with 0.4% glucose, Merck), and modified N-minimal media (MNMM, 5 mM KCl, 7.5 mM (NH_4_)2SO_4_, 0.5 mM K_2_SO_4_, 1 mM KH_2_PO_4_, 0.01 mM Tris–HCl pH 7.4, 1 mM MgCl_2_, 0.4% glucose, 38 mM glycerol, and 0.1% casamino acids).^45^

### Peptide Synthesis and Purification

The peptides were synthesized by a solid phase method on a rink amide MBHA resin using an ABI 433A automatic peptide synthesizer (Applied Biosystems). The resin-bound peptides (0.1 mequiv each) were cleaved from the resins by trifluoroacetic acid (TFA), washed with dry ether, and extracted with acetonitrile/water (50% v/v). All peptides were amidated at their C terminus. The peptides were further purified by reverse-phase high-pressure liquid chromatography (RP-HPLC) on a C_18_ reverse-phase Bio-Rad column (250 × 10 mm^2^, 300 Å pore size, 5 μm particle size). A linear gradient of 10–90% acetonitrile in water containing 0.1% TFA (v/v) in 40 min was used at a flow rate of 1.8 mL/min. Each crude peptide contained one major peak, as revealed by RP-HPLC. The purified peptides were shown to be homogeneous (>98%) by analytical HPLC. Electrospray mass spectroscopy confirmed their identity.^23^

### Fluorescent Labeling of the Peptides

Resin-bound peptides with a free N-terminal amino group were treated with rhodamine B *N*-hydroxysuccinimide dissolved in dimethylformamide (DMF) containing 3% diisopropyl ethyl amine for 24 h. The resin-bound rhodamine was then washed thoroughly with DMF and then with DCM, dried, and then cleaved using TFA. The peptides were purified (greater than 98% homogeneity) by RP-HPLC on a C_4_ column using a linear gradient of 10–90% acetonitrile in 0.1% TFA for 40 min. The peptides were subjected to amino acid and mass spectrometry analysis to confirm their composition.

### Construction of PmrAB Mutants

Mutants in the PmrAB system were created using the method of red recombinase.^1^ In short, *S. typhimurium* WT were transformed with the pKD46 plasmid after being passed in a *S. enterica* LB5010 restriction mutant. The bacteria were then grown in LB with ampicillin at 28 °C. Colonies were picked, grown in LB with ampicillin and 0.4% arabinose at 28 °C and made competent for electroporation using multiple washes in sterile distilled water with 10% glycerol and frozen at −80 °C until use. The DNA for knockout of the bacteria was composed of the gene for tetracycline together with its promoter, taken from pRS551, with 50bp flanking regions taken from the 5′ and 3′ of pmrA and pmrB accordingly (in *Salmonellae*, these genes are termed basS and basR). This fragment was inserted into a pSUPER plasmid, passed in a *S. enterica* LB5010 restriction mutant, and cut using restriction enzymes. The bacteria were then electrically transformed with the linear DNA product. The bacteria were grown for 2 h in SOC supplemented with 0.4% arabinose at 37 °C and then plated on SOC plates with tetracycline and grown at 37 °C. Colonies that grew on tetracycline but not on ampicillin were assayed using polymerase chain reaction (PCR) with one primer at the 5′ end of tetracycline and one at the 3′ end upstream of the inserted construct in the *Salmonellae* genome. PCRs of positive colonies were sent for sequencing to ensure that the pmrAB genes were replaced with tetracycline. The PmrAB knockout in the WT was termed PmrAB-KO, and the PmrAB knockout in the phoP-KO background was termed double-KO (DKO).

### Antimicrobial Activity of the Peptides

The antimicrobial activity of the peptides was examined in sterile 96-wells plates (Nunc F96 microtiter plates) in a final volume of 100 μL as follows. Aliquots (50 μL) of an overnight suspension-containing bacteria (late-log phase diluted 1:5000) were added to 50 μL of modified N-minimal media (MNMM) containing the peptides in a serial twofold dilution, and antibiotics, if required, to select for the specific mutant. Inhibition of growth was determined by the visibility of turbidity in comparison to blank with no growth after an incubation of 18–20 h at 37 °C with agitation. Antibacterial activity was expressed as the minimal inhibitory concentration (MIC) in which no growth could be observed.

### Extent of Membrane Polarization and Increase in Membrane Permeability of the Various Bacterial Strains

Typhimurium ATCC 14028 WT and the mutants were grown to the mid-log phase, centrifuged, and suspended in sodium phosphate buffer (SPB) (8 mM Na_2_HPO_4_, 2 mM NaH_2_PO_4_, pH = 7.4). Their OD was measured, and they were diluted to reach OD_600nm_ = 0.1 in 10 mL. Two microliters of SYTOX Green (for membrane permeability, Invitrogen, S7020) or 2 μL of 3,3′-dipropylthiadicarbocyanine-iodide [DiSC_2_(5), for membrane potential, Molecular Probes (Junction City, OR)] was added to the bacterial stock to a final concentration of 1 μM, and they were incubated with agitation for 10 min (membrane permeability) or 20 min (membrane potential) at room temperature. The bacteria were transferred to a black 96-wells plate (Nunc) (containing an AMP at different concentrations for membrane penetration), and a kinetic measurement was performed over 20 min using a BioTek multiplate reader (Synergy HT) fluorescence detector (SYTOX Green: ex = 486 nm, em = 528 nm; DisC_2_(5): ex = 620 nm, em = 680 nm).

### LPS Extraction and Purification

The cells for LPS isolation were grown in 1 L LB medium supplemented with 1 mM MgCl_2_ and the appropriate antibiotic at 37 °C until OD_600nm_ = 0.8 and subsequently harvested (7000 rpm, 15 min, room temperature). The pellet was resuspended in 10 mL of di-distilled water (DDW) and lyophilized. LPS isolation was performed according to the protocol of Darveau and Hancock.^46^ This method is based on the mechanical disruption of the cells, followed by sodium dodecyl sulfate (SDS) solubilization and magnesium precipitation of the LPS in cold ethanol. For cell breakage, the Precellys tissue homogenizer and the Precellys glass kit 0.1 mm (Peqlab Biotechnologie GmbH, Erlangen, Germany) were used instead of a French pressure cell. The yield of LPS obtained was quantified by the amount of 2-keto-3-deoxyoctonate (KDO) recovered relative to the cell dry weight.^47^ KDO purchased from Sigma was used for a standard curve. For visualization, 5 μg of LPS was loaded on SDS-polyacrylamide gel electrophoresis (PAGE) (15% resolving gel, 4% stacking gel) and stained using the sensitive silver staining method of Tsai and Frasch.^48^

### Lipid-A Isolation and Matrix-Assisted Laser Desorption/Ionization Time-of-Flight (MALDI-TOF) Analysis

To detect modifications in the lipid-A structure, lipid-A was isolated from LPS by mild hydrolysis, as described previously.^49^ All matrix-assisted laser desorption/ionization time-of-flight (MALDI-TOF) analyses were carried out in the negative-ion mode on a Bruker Biflex III MALDI-TOF (Bruker Daltonics, Bremen, Germany). Herein, we used the AB SCIEX 5800 MALDI-TOF/TOF System equipped with a Nd:YAG (355 nm) laser with 1 kHz pulse in a negative-ion mode to analyze the lipid-A composition of the different strains. Briefly, 0.5–1 mg of purified LPS was dissolved in 500 μL of 1% SDS in 10 mM sodium acetate buffer (pH 4.5) and incubated in an ultrasonic bath for 10 min. The samples were hydrolyzed at 100 °C for 1 h and then dried in a SpeedVac vacuum concentrator. The samples were washed with 100 μL of distilled water and 500 μL of acidified ethanol (100 μL of 4 M HCl was mixed with 20 mL of 95% ethanol) and centrifuged (2000*g*, 10 min, room temperature). The pellets obtained were washed again with 500 μL of 95% ethanol. After repeating the washing steps, lipid-A was finally resuspended in 100 μL of distilled water and lyophilized. As a matrix, we used 20 mg/mL 5-chloro-2-mercaptobenzothiazole (CMBT, Sigma-Aldrich) dissolved in methanol–chloroform (1:1). Typically, 300 shots were accumulated for each mass spectrum. The spectra were calibrated externally using the quantified peptides used as peptide standard (Bruker Daltonics).

### Peptide Binding to Whole Bacteria

The *Salmonella* WT and mutants were grown to mid-log in LB with the appropriate antibiotics, washed twice with phosphate-buffered saline (PBS), and diluted to OD_600nm_ = 1 in PBS. Rhodamine-labeled peptides were prepared at 3 μM, and 20 μL was dispensed to each tube. Each strain (180 μL) was added to the tubes to complete the volume to 200 μL with the peptides at a final concentration of 0.3 μM. The bacteria were incubated for 10–15 min and centrifuged at 14 000 rpm for 5 min. Fifty microliters from the supernatant was added to 50 μL of guanidinium–HCl 6 M (with 20 mM ethylenediaminetetraacetic acid (EDTA), 50 mM Tris–HCl, pH 6.5), and the fluorescence was read using a BioTek multiplate reader (Synergy HT) fluorescence detector (ex = 529 nm, em = 590 nm). The percentage of binding was calculated using tubes with the peptides but without bacteria for 0% binding. Treatments were done in triplicate.

### Atomic Force Microscopy Image Acquisition

AFM imaging was performed as described previously.^50^ Prior to the measurements, bacterial culture was grown to the mid-log phase, washed two times in PBS, resuspended in PBS, and adjusted to OD_600nm_ of 0.1. Bacteria were incubated for 4 h on freshly cleaved mica sheets coated with poly-l-lysine (0.01 mg/mL, P4832, Sigma) and fixated with 2% glutaraldehyde for 20 min. The samples were washed again with filtered DDW to remove glutaraldehyde traces and left to dry overnight at room temperature. Images of bacteria were acquired with MultiMode AFM (Bruker, Santa Barbara, CA) equipped with a NanoScope V controller and a small scanner. Images were recorded in air, at room temperature (22–24 °C), in the PeakForce quantitative nanomechanical mapping (QNM) mode using silicon nitride WS-levers (ORC8-PS-W, Olympus) with a nominal spring constant of 0.76 N/m. The PeakForce QNM AFM imaging mode yields quantitative nanomechanical mapping of material properties, including DMT modulus of elasticity (rigidity). At the same time, sample topography was imaged with high resolution (1024 pixels) and minimized sample distortion due to the fine adjustment of the force applied to the sample surface. The applied force was adjusted around 1 nN, and the scan rate was set to 1 Hz. Imaging was carried out at different scales to verify the consistency and robustness of the evaluated structures. Numerical data presented are the mean value (±standard deviation (SD)) of DMT modulus of four 150 × 150 nm^2^ samples, taken from a 700 × 700 nm^2^ field (*n* ≥ 8 cells for each treatment).

### AFM Image Analysis

The PeakForce QNM (Bruker, Santa Barbara, CA) mode enables acquiring topography images with elasticity mapping simultaneously. Representative images are presented in Figure 4. The topography of the bacterial cell surface is shown in Figure 4A. In addition, the estimations of bacterial cell length were done as distances between the left and right inflection points in the profiles taken, along the cell (Figure 4C). The elasticity of the bacterial cell wall was evaluated as the mean over a certain area at the DMT modulus map (Figure 4B). The areas for analysis were chosen to be the highest and most uniform areas possible (black rectangles). This was done to avoid the influence of cell surface geometry (cell surface curvature) on the measured values. All measurements were carried out with the original “NanoScope Analysis” software (Bruker, Santa Barbara, CA). Elements of flooding analysis (Figure 4D) were determined using WSxM software.^51^

### Transmission Electron Microscopy

The cells were centrifuged, and the pellet was loaded on aluminum disks with a depth of 100 μm (Engineering Office M. Wohlwend GmbH, Switzerland) and covered with a flat disc. The sandwiched sample was frozen in an HPM010 high-pressure freezing machine (Bal-Tec, Liechtenstein). The cells were subsequently freeze-substituted in an AFS2 freeze substitution device (Leica Microsystems, Austria) in anhydrous acetone containing 2% glutaraldehyde and 0.2% tannic acid osmium tetroxide for 3 days at −90 °C and then warmed up to −30 °C over 24 h. The samples were washed three times with acetone, incubated for 1 h at room temperature with 2% osmium tetroxide, washed three times with acetone, and infiltrated for 5–7 days at room temperature in a series of increasing concentrations of Epon in acetone. After polymerization at 60 °C, 60–80 nm sections were stained with uranyl acetate and lead citrate and examined in a Tecnai T12 electron microscope (FEI, Holland) operating at 120 kV, utilizing a 2k × 2k ES500W Erlangshen CCD camera (Gatan, U.K.).

## Results

### Effect of PhoPQ and PmrAB on the Susceptibility of the Bacteria to AMPs

We investigated both natural and *de novo*-designed AMPs (Table 1). The list includes (i) the human cathelicidin LL-37, (ii) a *de novo*-designed 15-mer AMP designated K_6_L_9_ and (iii) its d,l-amino acid-containing analogue, d,l-K_6_L_9_, and (iv) d,l amino acid histidine derivative, d,l-H_6_L_9_, (v) a 12-mer d,l-K_5_L_7_ and (vi) its caprylic-acid-conjugated analogue C8-d,l-K_5_L_7_, and (vii) the frog antimicrobial peptide Temporin L.^23^ These peptides have different net positive charges under the experimental conditions, as well as hydrophobicity (represented by RP-HPLC retention times), as indicated in Table 1. The peptides were tested for their antibacterial activity against WT *S. enterica* serovar Typhimurium ATCC 14028 (*S. typhimurium*) and its mutants (Table 2). The minimal inhibitory concentration (MIC) was defined using a broth microdilution antimicrobial assay. *S. typhimurium* have been widely used for the exploration of bacterial resistance acquired from these two TCSs.^52,53^ To determine whether a short-term induction would increase the resistance of the bacteria toward the peptides, which does not affect bacterial growth, we grew the bacteria in the presence of the peptides at declining concentrations and measured absorbance (*A*_600_) for cell growth. As shown in Table 2, phoP-KO was more susceptible to all AMPs compared to the WT and showed a similar susceptibility to the C8-K_5_L_7_ lipopeptide. Surprisingly, the pmrAB-KO gained resistance to four out of the seven peptides compared to the WT. However, similar to the phoP-KO, it showed a higher susceptibility to the histidine-substituted AMP. Interestingly, the double knockout (DKO) bacteria showed a susceptibility picture that combines the susceptibility picture of both single mutants. These results are probably attributed to the overall difference between the biophysical properties of the WT compared to the mutants and their susceptibilities toward the peptides.

**Table 1:** Peptides Used in This Study, Their Sequences, Net Charge, and RP-HPLC Retention Times

peptide designation	sequence^a^	net charge	relative hydrophobicity^b^ (% ACN)	retention time (min.)
LL-37	LLGDFFRKSKEKIGKEFKRIVQRIKDFLRNLVPRTES	+7	78.2	34.1
all l-K_6_L_9_	LKLLKKLLKKLLKLL	+7	77.6	33.8
d,l-K_6_L_9_	LK**L**LK**K**L**LK**KLL**K**LL	+7	65.4	27.7
d,l-K_5_L_7_	KK**LL**KLL**L**K**L**LK	+6	57	23.5
C_8_-d,l-K_5_L_7_	CH_3_(CH_2_)_6_CO-KK**LL**KLL**L**K**L**LK	+5	68.2	29.1
Temporin L	FVQWFSKFLGRIL	+3	74.6	32.3
d,l-H_6_L_9_	LH**L**LH**H**L**LH**HLL**H**LL	+1	68.6	29.3

a All of the peptides are amidated at their C-termini. Underlined and bold font amino acids are d-enantiomers.

b Relative hydrophobicity is reflected by the percent of acetonitrile at the retention time.

**Table 2:** MIC Value (μM) of the Various Peptides on the Bacteria Studied^a^

peptide designation	WT	phoP-KO	pmrAB-KO	DKO
LL-37	3.125	1.56	6.25	3.125
l-K_6_L_9_	1.56	0.39	3.125	3.125
d,l-K_6_L_9_	1.56	0.39	1.56	0.78
d,l-K_5_L_7_	6.25	1.56	12.5	3.125
C8-d,l-K_5_L_7_	3.125	3.125	6.25	3.125
Temporin L	25	12.5	25	25
d,l-H_6_L_9_	100	50	50	50

a WT: *S. enterica* serovar Typhimurium 14 028s wild-type phoP-KO; *S. enterica* serovar Typhimurium 14 028s phoP knockout pmrAB-KO; *S. enterica* serovar Typhimurium 14 028s PmrAB knockout DKO; *S. enterica* serovar Typhimurium 14 028s phoP and PmrAB knockout.

### Bacterial Membrane Polarization

**Figure 1:**
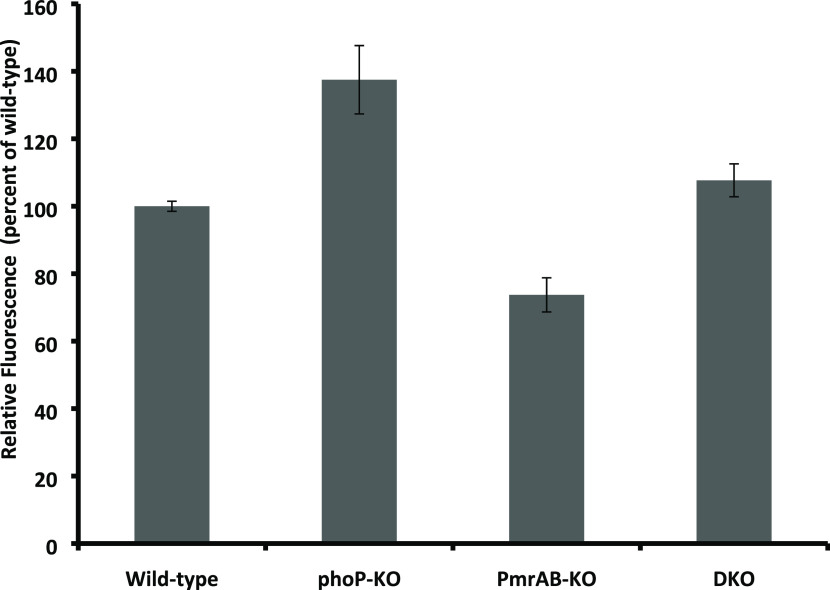
Transmembrane potential of the WT and mutant *Salmonellae*. The WT and mutant *Salmonellae* were grown to the mid-log growth phase and then washed twice with SPB. Then, the bacteria were diluted to OD_600nm_ = 0.1, and DisC_2_(5) dye was added (1 μM final concentration). The bacteria were incubated with the dye for 20 min, and then the fluorescence was measured (ex = 620 nm, em = 680 nm). All data represent mean ± SD from three independent experiments. Error bars represent standard error. One-way analysis of variance was used to analyze the data. Results showed a statistically significant difference (*P-value <0.005).

We examined the effect of the TCSs on the ability of the bacteria to maintain membrane potential using the potential-sensitive dye DisC_2_(5), whose fluorescence is quenched due to the oligomerization of the dye upon binding to the membrane of electrically polarized cells. The data shown in Figure 1 reveal that both WT and pmrAB-KO are more polarized compared to phoP-KO and DKO bacteria, albeit not strongly. This suggests that the membranes of the WT and pmrAB-KO are less permeable compared to the membranes of the other two mutants. In agreement with these results, the antimicrobial activities (Table 2) of most peptides toward the WT and the pmrAB-KO mutant are similar but lower compared to phoP-KO and DKO, both of which also share similar susceptibilities to most of the peptides. These results also agree with the higher rigidity of the WT and the pmrAB-KO compared to phoP-KO and DKO (see the sections on AFM studies)

### Permeability of the Bacteria to SYTOX Green

**Figure 2:**
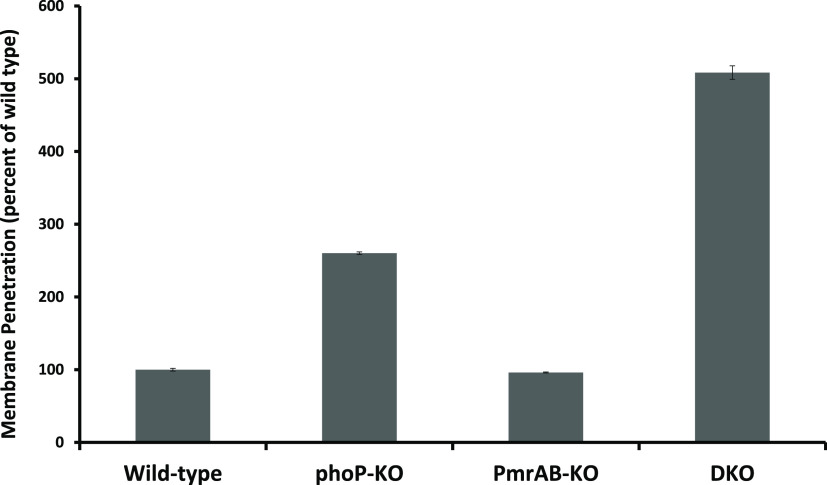
Membrane penetration assay of the WT and mutant *Salmonellae*. The WT and mutant *Salmonellae* were grown to the mid-log growth phase and then washed twice with SPB. The bacteria were diluted to OD_600nm_ = 0.1, and SYTOX Green dye was added (1 μM final concentration). The bacteria were incubated with the dye for 10 min. Then, the bacteria were added to a black opaque 96-wells plate containing C8-d,l-K_5_L_7_ at different concentrations. The fluorescence was measured (ex = 485 nm, em = 529 nm) after 3 min of incubation. All data represent mean ± SD from three independent experiments. Error bars represent standard error. One-way analysis of variance was used to analyze the data. Results showed a statistically significant difference (*P < 0.001).

The fluorescent dye SYTOX Green was used to observe whether the KO of the TCSs has an effect on the permeability of the bacterial membrane. The fluorescence of this dye increases upon binding to nucleic acids, thus indicating DNA binding and cell penetration. The results shown in Figure 2 represent the percentage of fluorescence compared to the WT, taken as 100%. The data reveal that the fluorescence of the WT and the pmrAB-KO mutant is considerably less than that of the phoP-KO and DKO.

### Binding Affinity of Peptides

**Figure 3:**
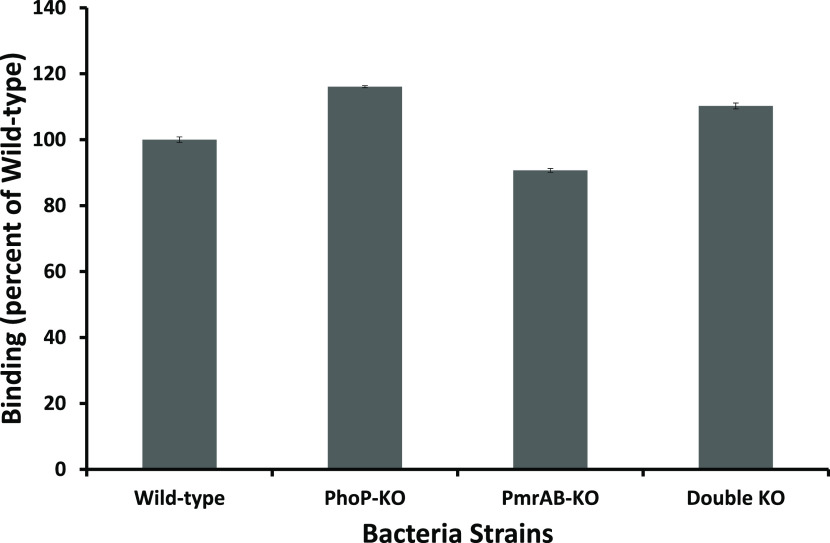
Binding of KKkKK-rhodamine to the bacteria. Bacteria were grown to the mid-log growth phase and diluted to OD_600nm_ = 1 in PBS (−/−). Then, the lysine-rhodamine peptide was added and incubated with the bacteria for 10 min. The bacteria were then centrifuged at 14 000 rpm, and the supernatants were transferred to a black 96-wells plate. The fluorescence of the supernatants was measured (ex = 529 nm, em = 590 nm) and compared to a control without bacteria to calculate the percent of binding. Results are an average of five independent experiments (*N* = 4 per experiment). Error bars represent standard error. One-way analysis of variance was used to analyze the data. Results showed a statistically significant difference (*P* < 0.002).

The differences in the activities of the peptides on the bacteria could also be attributed to differences in their binding to the bacteria. This property is also influenced by the charge of the bacterial surface. To test this possibility, we synthesized and fluorescently labeled a highly positively charged peptide, pentalysine, with the fluorescent probe rhodamine. A d-amino acid was incorporated into the peptide to disrupt its conformation and eliminate the possible contribution of the structure to the binding process. The bacteria were sedimented by centrifugation, and the supernatant was sampled to measure the remaining fluorescence. The fluorescence of the supernatant was compared to that of a control without bacteria to calculate the percentage of binding. The data shown in Figure 3 reveal a similar extent of binding to WT and the pmrAB-KO, which is less than that of phoP-KO and DKO but are also similar to each other. Importantly, similar to the membrane polarization assay, these results correlate with the antimicrobial activity (Table 2) since most peptides have the same activity on the WT and the pmrAB-KO mutant, which is lower compared to phoP-KO and DKO that also share similar susceptibilities to most of the peptides.

### Transmission Electron Microscopy Images of the Bacteria

**Figure 4:**
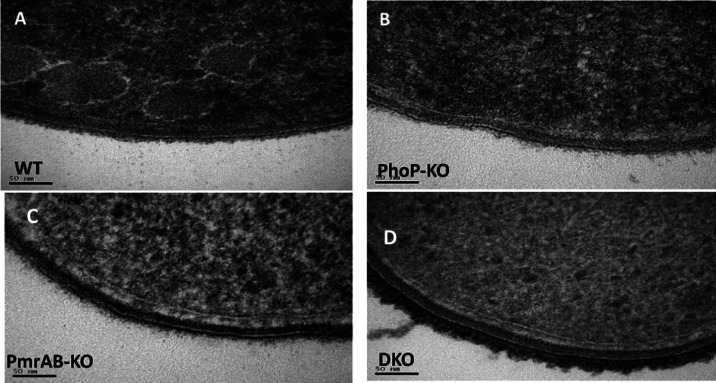
Imaging of the bacteria using transmission electron microscopy (TEM). Bacteria were imaged using TEM, and representative images were chosen. The two membranes of these Gram-negative bacteria can be seen as white thin lines, a stronger (darker) staining of the bacteria means a denser array of proteins. (A) WT bacteria revealed a single and uniform electron density staining at the periplasmic space and a thin layer of highly stained material over the outer membrane. (B) phoP-KO strain exhibited light staining of the membranes and almost absent surface elements. (C) pmrAB-KO strain displayed a layer structure with three distinct zones in its periplasmic space and a thick dark layer beyond the outer membrane. (D) DKO strain resembles that of the WT and phoP-KO strains but with thicker and longer external polysaccharides and surface elements attached to its surface.

**Figure 5:**
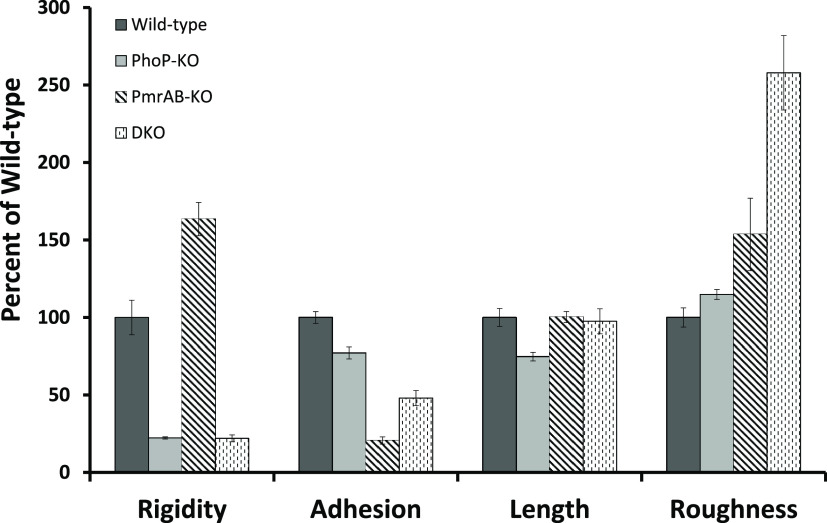
Comparison of the rigidities of WT and mutant *Salmonellae*. All values of rigidities are normalized to the WT (100%). Each bar represents the corresponding mean value over 10 cells. All data represent mean ± SD from three independent experiments. Error bars represent standard error. One-way analysis of variance was used to analyze the data. Results showed a statistically significant difference (*P-value <0.005).

Resistance to AMPs could result from a change in the structure of the cell wall as suggested for *Staphylococcus aureus*.^54^ High-pressure freezing and free substitution followed by transmission electron microscope (TEM) were therefore used to visualize cross sections of the bacteria, allowing us to visualize the (i) inner and outer membranes, (ii) periplasmic space, and (iii) cell wall thickness and density. Figure 4 shows the representative images of the WT and mutants. As expected, the cell wall of all of the bacteria contains two lipid layers seen as white lines. However, an intense electron opaque outer layer and a dense periplasmic layer are observed only in pmrAB-KO (Figure 4), which might explain the high rigidity of the cell wall of pmrAB-KO compared to the other strains (Figure 5).

The TEM pictures of the WT bacteria revealed a single and uniform electron density staining at the periplasmic space and a thin layer of highly stained material over the outer membrane, commonly attributed to surface-anchored proteins (Figure 4A). The phoP-KO strain exhibited light staining of the membranes and almost absent surface elements (Figure 4B). Remarkably, the pmrAB-KO strain displayed a layer structure with three distinct zones in its periplasmic space, and a thick dark layer beyond the outer membrane, caused by the high anionic charge of the LPS (Figure 4C). Interestingly, the staining pattern of the DKO strain resembles that of the WT and phoP-KO strains (Figure 4D) but with thicker and longer external polysaccharides due to the longer LPS chains and surface elements attached to its surface.

In addition, we found a substantial difference between the average thickness of the periplasmic thickness between WT (12.0 ± 1.0 nm) and that of phoP-KO (14.0 ± 1.5 nm) and to a much lesser extent with that of pmrAB-KO (20.0 ± 1.0 nm) and DKO mutants (20.0 ± 1.1 nm).

### Extraction and Analysis of the Lipopolysaccharides

**Figure 6:**
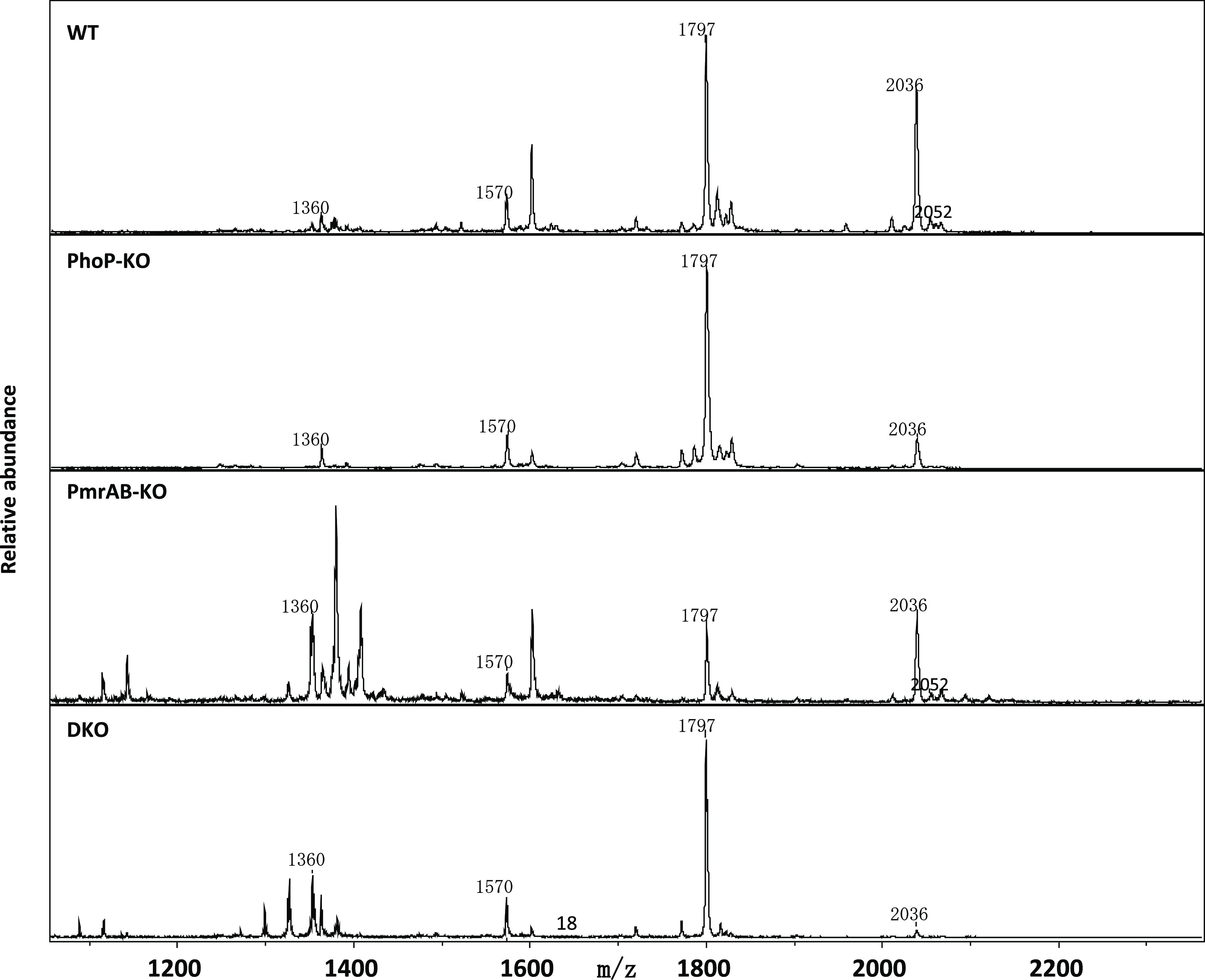
LPS analysis. MALDI-TOF mass spectra of purified lipid-A. The major signals represent the hepta-acylated form of lipid-A (*m*/*z* 2036) and the hexa-acylated form lacking palmitate (*m*/*z* 1797). Moreover, the addition of a hydroxyl group (*m*/*z* 2052) to the hepta-acylated lipid-A could be detected in the WT and in the pmrAB-KO.

Following the TEM results that showed changes in the cell wall, as well as the notion that the lipid composition of the LPS can be affected by these TCSs, the LPS portions of all four strains were isolated, and the lipid-A was analyzed (Figure 6). The major signals of the WT lipid-A represent the hexa-acylated form (*m*/*z* 1797) and the hepta-acylated form (*m*/*z* 2036), which is generated by the addition of a palmitate chain. Moreover, the hydroxylation of the hexa- and hepta-acylated lipid-A could be detected (*m*/*z* 1814, 2052). Both modifications are catalyzed by enzymes (PagP, LpxO) whose expression is regulated by the TCS PhoPQ.^55,56^ The peaks for aminoarabinose-substituted lipid-A were not evident in the WT because this substitution (mediated by PmrAB) occurs only under Mg^2+^-deficient conditions. Thus, the lipid-A of the pmrAB-KO strain generated a similar structural pattern as the WT in agreement with their similar susceptibility to most of the AMPs tested here (Table 2). In comparison, the lipid-A of the phoP-KO strain and DKO is similar and consists primarily of hexa-acylated lipid-A (*m*/*z* 1797), which is in agreement with their similar susceptibility to most of the AMPs (Table 2).

### Cell Wall Properties Determined by Single-Cell Atomic Force Microscopy

**Figure 7:**
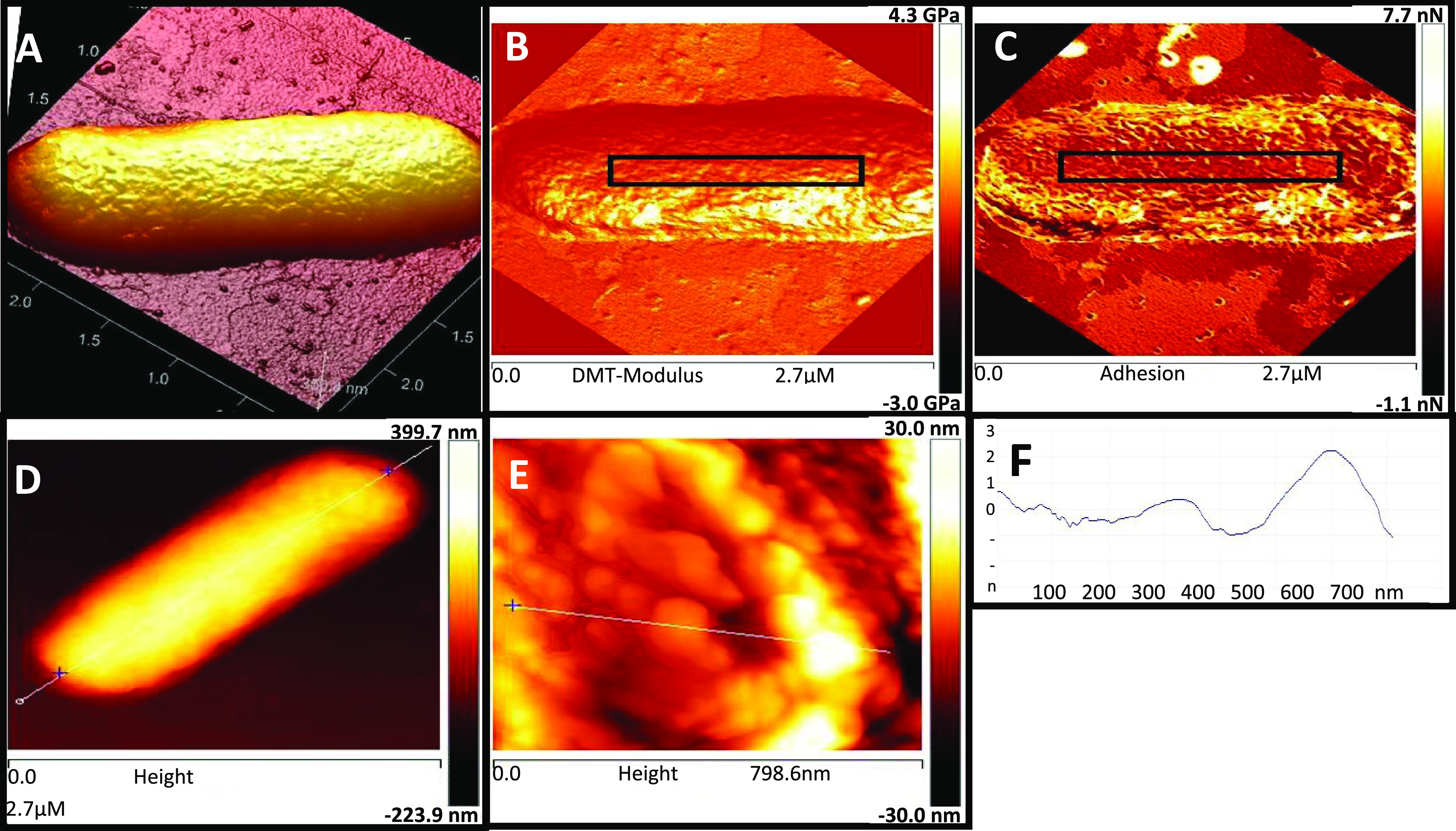
Imaging and measurements of bacterial cell characteristics by AFM. (A) Three-dimensional (3D) reconstruction of *S. typhimurium* bacterium on a mica substrate; (B, C) DMT modulus measurement (influenced by the thickness of the bacteria wall), measured as the mean value over the area marked by a black rectangle; (D) measurement of the length of the bacterium (white line), measured as the distance between the left and right inflection points with correction for the tip-sample convolution. (E) High-resolution image of the cell surface. Imaging and measurements were carried out by the PeakForce quantitative nanomechanical mapping (QNM) mode. (F) Cross section of (E) seen along the white line.

Atomic force microscopy (AFM) is a powerful tool not only for imaging the nanoscale features of bacteria but also for measuring the mechanical characteristics of their surfaces, such as rigidity.^57,58^ We explored these properties using the quantitative nanomechanical mapping (QNM) mode of AFM. In this mode, AFM performs multiple, fast indentations over the scanned surface, using a silicon probe, allowing the measurement of the tip-sample interaction. The shape of the indentation curves allows the evaluation of both the elastic response of the bacterial surface to the AFM probe indentation and the detachment force of the probe from the surface. It also allows the construction of the corresponding images (maps) of the cell surface simultaneously with regular topography images, allowing the measurement of geometrical characteristics such as length and height (Figure 7).

In such a fashion, randomly chosen bacterial cells were imaged, and a flat rectangular region in their maps was chosen for the construction of topography images (Figure 7A,C) and calculation of elastic modulus (Figure 7B). All data were normalized to WT values. Using this analysis, we found (Figure 4) that compared to the WT, the surface of the pmrAB-KO bacteria is much more rigid (181% of WT), whereas that of the phoP-KO bacteria is markedly less rigid (22% of WT).

**Figure 8:**
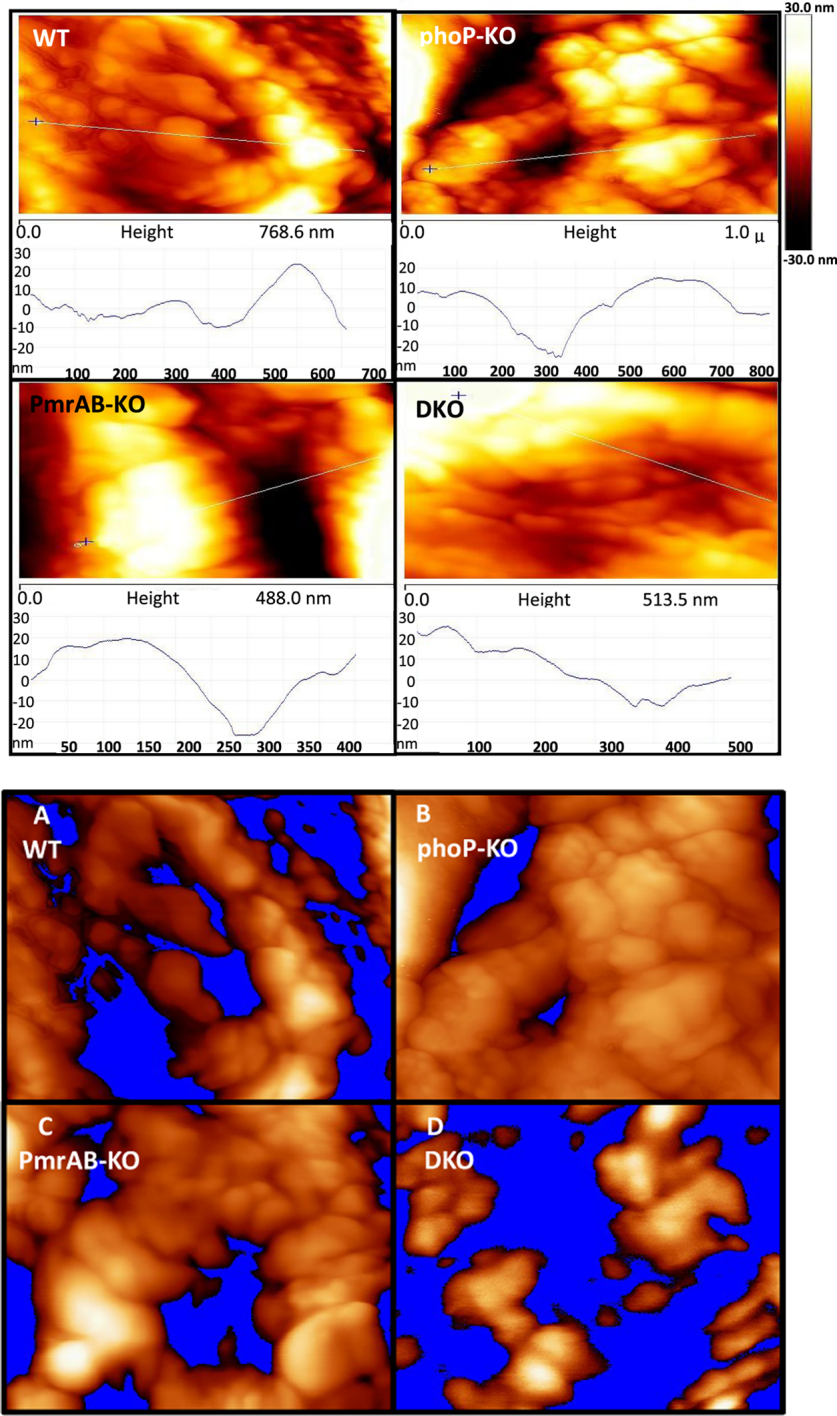
Flooding analysis of the bacteria using AFM. The traces shown above are height analyses along the white lines. Sample images shown below from the flooding analysis of WT (A), phoP-KO (B), pmrAB-KO (C), and double-KO (D) bacterial cells. The images exhibit surface topography above a certain height threshold. All topography features beneath this threshold are marked blue.

Two additional characteristics, connectivity and uniformity of bacterial surfaces, can be explored using a “flooding analysis”^51^ (Figure 8). This analysis compares the height uniformity of the bacterial surface elements. In this analysis, surface features appearing over a certain height threshold are kept, and features beneath that threshold are regarded as a uniform background (colored in blue). Using this approach, a cell surface featuring no holes or shallow holes would be regarded as uniform, whereas a surface with deep holes and dents would be regarded as nonuniform. A complex surface would be a surface featuring a lot of holes and elements with connectivity between them. Visual inspection of the flooding analysis images suggests that at a given threshold value (20 nm), WT exhibits uniform, but complex, possibly highly interconnected, protein–protein complex structures. This topography might be the result of the LPS and other surface elements protruding from the surface with protein complexes and channels scattered among them; phoP-KO bacteria, given a lower threshold value, exhibit an almost uniform surface with a few deep holes. This can be due to the fact that the phoP-KO membrane is thinner and more uniform, with less protruding surface elements. This finding is further emphasized in the TEM image of the phoP-KO bacteria (Figure 4). PmrAB-KO bacteria look more similar to the WT and exhibit a less uniform surface and correspondingly less complex connectivity. This might be due to tighter connections on the surface of the bacteria. The data also reveal that the DKO bacteria exhibit a nonuniform surface with very low connectivity, even for a 2-fold higher threshold compared to the one used for the WT. They also show higher ridges (lighter color). This means that the bacterial surface has long protruding elements scattered on it, which could be due to the uncontrolled polymerization of surface elements, as can be seen in the TEM picture (Figure 4). These protruding elements probably prevent the tip from penetrating into the lipidic membrane, which might give an unclear interpretation of the bacterial surface.

Overall, the data reveal similar connectivity and uniformity between the WT and PmrAB-KO in agreement with their susceptibility toward most of the peptides tested. However, due to the uncertainty in the data with the DKO (as explained above) and phoP-KO, it is not clear why both of them have a similar susceptibility to most of the AMPs.

## Discussion

The PhoPQ and PmrAB TCSs have been the focus of various studies concerning their potential to induce bacterial resistance to AMPs. These systems could be activated by AMPs, leading to the induction of enzymes that modify the outer surface of Gram-negative bacteria and make it less penetrable to AMPs. In line with that, it was shown that the deletion of the PhoPQ system increases the susceptibility of bacteria to AMPs.^23,39^ A few studies also reported the role of PmrAB TCS in resistance toward polymyxin B and colistin, both of which destabilize the architecture of the outer membrane through specific interactions with LPS.^23,59^ However, the biophysical aspects and the combined effects of PhoPQ and PmrAB TCSs, as well as their contribution to bacterial resistance to cationic AMPs, are yet not well known. We performed our study with *S. typhimurium*, a frequently used model bacterium, and investigated the roles of both PhoPQ and PmrAB systems on resistance to a diverse array of AMPs. The results were correlated with the changes in the biophysical properties of the bacteria. For that purpose, we created mutants deficient in the individual systems including a DKO mutant. The lack of these systems indeed changed the composition of the bacterial LPS, as confirmed by mass spectrometry (Figure 6).

Using a biophysical approach we explored the effects of the two systems on several characteristics of the bacteria, which in turn have an impact on the antimicrobial activity of AMPs. These characteristics are (i) bacterial membrane polarization and permeation, (ii) surface charge, and (iii) cell wall rigidity and density.

### Effect of the Mutations on the Extent of Bacterial Membrane Polarization and Permeability

Examination of the extent of bacterial cell polarization reveals that the phoP-KO and DKO bacteria are similarly polarized and both are less polarized than the WT and the pmrAB-KO (Figure 1). It has been demonstrated that the rigidities of phoP-KO and DKO are significantly less than that of the WT, In contrast, the pmrAB-KO bacteria are more rigid than the WT, which indicates that the phoP-KO and DKO cell walls are more permeable than the WT and the pmrAB-KO, which is in agreement with the higher activity of most of the peptides on the phoP-KO bacteria compared to the pmrAB-KO.

Further support for the differences in membrane permeability comes from the SYTOX experiments in which we measure the extent of membrane permeability. The data showed a trend that is similar to that of the membrane polarizing experiment, *i.e.*, the WT and pmrAB-KO are less permeable than phoP-KO and the DKO (Figure 2). This is in line with changes in the composition of the bacterial cell wall components of the different bacterial strains due to the deletion of the TCSs, as revealed by mass spectrometry of the LPS isolated from all of the bacteria (Figure 5). These results are also in agreement with the MICs of most of the peptides on the different bacteria, showing a direct correlation between permeability and susceptibility to AMPs.

### Effect of the Mutations on the Surface Charge

The PmrAB system is known to activate the pmrHFIJKLM operon, which synthesizes and adds 4-aminoarabinose (l-Ara4N) to the LPS, making the surface less negatively charged.^60,61^ Because there is communication between the two systems through pmrD, the PhoPQ system can also contribute to such an effect on the surface charge of the bacteria.^29,62^ This effect should reduce the binding of the positively charged AMPs to the surface, making the bacteria more resistant. Indeed, as expected, phoP-KO and DKO bacteria bind similarly to the pentapeptide to a higher extent than the WT bacteria. However, the pmrAB-KO has a binding capacity similar to the WT (Figure 3), the reason for which is not yet clear. Nevertheless, as seen with the other assays, the WT and pmrAB-KO are similarly sensitive to most AMP. Overall, the extent of peptide binding to the various bacteria is directly correlated to their susceptibility to AMPs.

### Bacterial Biophysical Properties: Surface Density, Rigidity, and Thickness of the Periplasmic Space

The third factor that could contribute to the resistance of bacteria to AMPs relates to the biophysical properties of the bacteria, which include cell wall density and rigidity. The PhoPQ and PmrAB systems are known to induce enzymes that add and remove fatty acids from the LPS^63,64^ and also modify the length of the sugar residues of the LPS.^53,65^ This suggests that the activation of the two systems can affect the physical properties of the bacterial surface. To examine this possibility, we used both TEM and AFM techniques. The TEM images of the cross sections of the WT and the mutated bacteria shown in Figure 4 clearly reveal that the outer membrane of pmrAB-KO bacteria is stained much stronger than those of the WT, phoP-KO, and DKO bacteria. This suggests that the pmrAB-KO outer surfaces are denser compared to the other three, possibly due to the chelation of metal ions that replaced uranyl acetate during sample preparation. This can explain why the pmrAB-KO bacteria are not as sensitive as the phoP-KO and DKO to the AMPs investigated.

It has been revealed from the electron microscopic results that the thickness of the periplasmic layer of the phoP-KO bacteria is significantly smaller than that of the WT (69% of WT) and those of pmrAB-KO and DKO to a lesser extent (86% of WT). These properties can also contribute to the similarities in the susceptibility of phoP-KO and DKO. Regarding pmrAB-KO, its thinner periplasmic space compared to WT can compensate for its high rigidity, and therefore it has susceptibility to AMPs similar to that of the WT. We also cannot rule out the possibility that the PmrAB system affects membrane fluidity, and when it is deficient, the membrane is denser and more rigid and thus stains stronger. The AFM studies further demonstrated significant differences between the rigidities of the various bacteria (Figures 7 and 8). The data reveal that the rigidities of phoP-KO and DKO are significantly less than that of the WT (22 and 67%, of WT, respectively). In contrast, the pmrAB-KO bacteria are more rigid than the WT (180% of WT). This result is consistent with our previous assays suggesting that the lack of the PhoPQ system makes the bacterial surface less dense and more penetrable to AMPs, while the lack of the PmrAB system makes the surface more rigid and less penetrable.

In summary, the PhoPQ and PmrAB TCSs have opposing effects on the biophysical properties and biological activities of the bacteria. The phoP-KO bacteria are more susceptible to most AMPs, have a less rigid surface, are more penetrable, and their surface is more negatively charged compared to WT. In contrast, the pmrAB-KO bacteria preserved the activity of a range of AMPs, have a very dense and rigid cell wall, and are less penetrable. This suggests that the PhoPQ system contributes to the strengthening of the bacterial cell wall, which helps them defend AMPs, possibly just making it more fluid and malleable. In contrast, activation of the PmrAB system has a role in the degeneration of the cell wall, thus making the bacteria more sensitive to AMPs.

Overall, the data suggest that the coexistence of systems with opposing effects on the biophysical properties of the bacteria contribute to their membrane flexibility, which is important to accommodate changing environments but inhibits the development of meaningful resistance.

### Statistical Analysis

The results were analyzed using a one-way analysis of variance and compared with the WT. Values of *P* < 0.005 were considered statistically significant.
